# Meta-analysis of biomarkers for severe dengue infections

**DOI:** 10.7717/peerj.3589

**Published:** 2017-09-15

**Authors:** Kuan-Meng Soo, Bahariah Khalid, Siew-Mooi Ching, Chau Ling Tham, Rusliza Basir, Hui-Yee Chee

**Affiliations:** 1 Department of Microbiology and Parasitology, Faculty of Medicine and Health Sciences, Universiti Putra Malaysia, Serdang, Selangor, Malaysia; 2 Department of Medicine, Faculty of Medicine and Health Sciences, Universiti Putra Malaysia, Serdang, Selangor, Malaysia; 3 Malaysian Research Institute on Ageing, Universiti Putra Malaysia, Serdang, Selangor, Malaysia; 4 Department of Biomedical Science, Faculty of Medicine and Health Sciences, Universiti Putra Malaysia, Serdang, Selangor, Malaysia; 5 Department of Human Anatomy, Faculty of Medicine and Health Sciences, Universiti Putra Malaysia, Serdang, Selangor, Malaysia

**Keywords:** dengue, Cytokine, Chemokine, Severity, Biomarkers

## Abstract

**Background:**

Dengue viral infection is an acute infection that has the potential to have severe complications as its major sequela. Currently, there is no routine laboratory biomarker with which to predict the severity of dengue infection or monitor the effectiveness of standard management. Hence, this meta-analysis compared biomarker levels between dengue fever (DF) and severe dengue infections (SDI) to identify potential biomarkers for SDI.

**Methods:**

Data concerning levels of cytokines, chemokines, and other potential biomarkers of DF, dengue hemorrhagic fever, dengue shock syndrome, and severe dengue were obtained for patients of all ages and populations using the Scopus, PubMed, and Ovid search engines. The keywords “(IL1* or IL-1*) AND (dengue*)” were used and the same process was repeated for other potential biomarkers, according to Medical Subject Headings terms suggested by PubMed and Ovid. Meta-analysis of the mean difference in plasma or serum level of biomarkers between DF and SDI patients was performed, separated by different periods of time (days) since fever onset. Subgroup analyses comparing biomarker levels of healthy plasma and sera controls, biomarker levels of primary and secondary infection samples were also performed, as well as analyses of different levels of severity and biomarker levels upon infection by different dengue serotypes.

**Results:**

Fifty-six studies of 53 biomarkers from 3,739 dengue cases (2,021 DF and 1,728 SDI) were included in this meta-analysis. Results showed that RANTES, IL-7, IL-8, IL-10, IL-18, TGF-b, and VEGFR2 levels were significantly different between DF and SDI. IL-8, IL-10, and IL-18 levels increased during SDI (95% CI, 18.1–253.2 pg/mL, 3–13 studies, *n* = 177–1,909, *I*^2^ = 98.86%–99.75%). In contrast, RANTES, IL-7, TGF-b, and VEGFR2 showed a decrease in levels during SDI (95% CI, −3238.7 to −3.2 pg/mL, 1–3 studies, *n* = 95–418, *I*^2^ = 97.59%–99.99%). Levels of these biomarkers were also found to correlate with the severity of the dengue infection, in comparison to healthy controls. Furthermore, the results showed that IL-7, IL-8, IL-10, TGF-b, and VEGFR2 display peak differences between DF and SDI during or before the critical phase (day 4–5) of SDI.

**Discussion:**

This meta-analysis suggests that IL-7, IL-8, IL-10, TGF-b, and VEGFR2 may be used as potential early laboratory biomarkers in the diagnosis of SDI. This can be used to predict the severity of dengue infection and to monitor the effectiveness of treatment. Nevertheless, methodological and reporting limitations must be overcome in future research to minimize variables that affect the results and to confirm the findings.

## Introduction

Dengue virus infection is a well-known worldwide health problem. The spectrum of clinical manifestations can rapidly develop into its most severe form, dengue shock syndrome (DSS), which has the worst outcome despite aggressive standard of care management. Dengue infection had a fatality rate between 0.2% and 0.6% in Malaysia between the years 2000 and 2014 (Ministry of Health Malaysia, 2015). Malaysia has a national target to reduce this fatality rate to less than 0.2%; therefore, there is a need to find suitable severity markers for this disease.

Previous studies have proposed mechanisms that lead to severe symptoms. In vitro studies have found no structural damage or cell death in infected endothelial cells (Chaturvedi et al., 2000; Srikiatkhachorn et al., 2006). Moreover, differences in levels of cytokines and chemokines were found when sera/plasma samples from dengue hemorrhagic fever (DHF) and dengue fever (DF) patients were compared (De la Cruz Hernández et al., 2016), which suggests that severe symptoms are caused by the cytokines and chemokines that act as mediators and vasodilators in blood, rather than by direct virus-induced cell damage. This hypothesis also explains the observation that severe symptoms occur during defervescence, after the decrease of viremia (Dejnirattisai et al., 2008). Addtionally, the transient quality of cytokines and chemokines also explains the short lived and rapid recovery nature of severe symptoms found in patients, if monitored carefully. As cytokines, chemokines, and other biomarkers play important roles in the mechanism of dengue infection, they could be potential markers of severity.

To predict the progression of dengue infection, the World Health Organization (WHO) classified thrombocytopenia, petechiae, and low hematocrit as predictive symptoms in 1997. In 2009, WHO revised these criteria as they were poorly associated with severe dengue (SD) cases (Azeredo et al., 2006; Horstick & Ranzinger, 2015). Several clinical symptoms were listed as warning signs of SD infections (SDI) in 2009; however, to date, there is no routine laboratory biomarker with which to predict the disease progression of dengue infection (Srikiatkhachorn & Green, 2010). In this context, numerous studies (Kelley, Kaufusi & Nerurkar, 2012; Reis et al., 2007; Seet et al., 2009; Tay & Tan, 2006) and reviews (Chaturvedi et al., 2000; Huy et al., 2013; John, Lin & Perng, 2015; Page & Liles, 2013; Yacoub & Wills, 2014) have been conducted to propose potential severity biomarkers. Despite this, one of the major hindrances of this effort is the inconsistency of results caused by heterogeneity among the studies. Biomarker levels have been found to be affected by factors such as timing of sample collection, processing of samples into plasma or serum, WHO classification method used to assign the disease’s severity, host’s immune status, and dengue serotypes (Srikiatkhachorn & Green, 2010). This study therefore aims to identify potential severity biomarkers and to study the factors causing inconsistency.

## Methods

### Literature search

A systematic review of mean difference in biomarker levels between DF and SD infections was undertaken. This was based on the principles recommended in the Preferred Reporting Items for Systematic Reviews and Meta-analyses (PRISMA) statements.

#### Data source

Relevant articles were identified via a systematic search of the MEDLINE (1946—present; via Ovid), non-indexed citations (via Ovid), Embase (1974—present; via Ovid), PubMed, and Scopus databases. The reference lists of published reviews were also manually screened to retrieve more relevant articles. Only articles published in English were evaluated.

#### Search strategies

A search of *in vivo* studies was carried out from October 12, 2015–October 12, 2016, using subject headings and free text terms. Search engines were utilized with specific keywords, as listed in Table 1. These keywords were Medical Subject Headings (MESH) terms suggested by PubMed and Ovid.

**Table 1:** List of keywords used.

Types of proteins	Keywords
Interleukin (IL)	(IL1* OR IL-1*) OR (IL2* OR IL-2*) OR (IL3* OR IL-3*) OR (IL4* OR IL-4*) OR (IL5* OR IL-5*) OR (IL6* OR IL-6*) OR (IL7* OR IL-7*) OR (IL8* OR IL-8*) OR (IL9* OR IL-9*) OR (IL10* OR IL-10*) OR (IL11* OR IL-11*) OR (IL12* OR IL-12*) OR (IL13* OR IL-13*) OR (IL14* OR IL-14*) OR (IL15* OR IL-15*) OR (IL16* OR IL-16*) OR (IL17* OR IL-17*) OR (IL18* OR IL-18*) OR (IL19* OR IL-19*) OR (IL20* OR IL-20*) OR (IL21* OR IL-21*) OR (IL22* OR IL-22*) OR (IL23* OR IL-23*) OR (IL24* OR IL-24*) OR (IL25* OR IL-25*) OR (IL26* OR IL-26*) OR (IL27* OR IL-27*) OR (IL28* OR IL-28*) OR (IL29* OR IL-29*) OR (IL30* OR IL-30*) OR (IL31* OR IL-31*) OR (IL32* OR IL-32*) OR (IL33* OR IL-33*) OR (IL34* OR IL-34*) OR (IL35* OR IL-35*) OR (IL36* OR IL-36*) AND (dengue*)
Chemokine (C-C motif) ligand, (CCL) and Chemokine (C-X-C motif) ligand (CXCL)	(MCP*) OR (MIP*) OR (RANTES*) OR (Eotaxin*) OR (C-TACK*) OR (CXCL9*) OR (CXCL10*) OR (IP10* OR IP-10) OR (CXCL11*) OR (SDF1*) AND (dengue*)
Others	(TNF*) OR (MIF*) OR (TGF*) OR (ST-2* OR ST2) OR (selectin*) OR (VEGF*) OR (VCAM*) OR (ICAM*) OR (IFN*) OR (TRAIL*) OR (MMP*) AND (dengue*)

### Inclusion criteria

Cross-sectional and cohort studies concerning biomarker levels in dengue patients from all age groups and regions were included, regardless of publication year.

### Exclusion criteria

The relevance of each paper was determined based on the type of article. Reviews that did not contain original research data were excluded. Nonetheless, the reference lists of reviews were manually screened to retrieve more relevant articles. In addition to reviews, proceedings that did not employ the peer-review process were also excluded. Beyond that, the objectives of papers were evaluated and objectives that did not involve human patients were excluded. Studies that did not involve the infection of the whole virus (for example, vaccine research involving only recombinant proteins from virus) were also excluded, as were studies that involved complications with other diseases. Finally, the data for each paper were assessed. As dengue viral infection is acute, this research did not include data collected more than 20 days after the onset of fever, to reduce data heterogeneity.

Furthermore, papers that did not present data separately for DF and SDI were excluded. In addition, papers that did not provide the number of blood samples, the means and standard deviations of biomarker levels, or the raw data that allowed the calculation of these parameters were also omitted. Nevertheless, citations in these papers were recorded in Sheet 11 of  Supplemental Information 2. The authors of these papers were contacted to obtain necessary unpublished data.

### Data abstraction

#### Study selection

Potentially relevant studies from the literature search were identified by screening the type of article, the title, and the abstract. This screening was conducted by one reviewer and was further confirmed by a second reviewer. After excluding duplicates, studies without abstract, and apparently irrelevant studies, the full text of the remaining studies were screened for relevant data by two reviewers. Disagreements between the reviewers were resolved through consensus.

#### Data extraction

The following data was extracted independently and in duplicate by two reviewers (KM Soo and SM Ching): citations in the study, participant characteristics (population and age), study duration, time interval of the experiment expressed as range of days of fever, name of measured biomarkers, WHO classification method, mean and standard deviation of biomarker levels, number of samples, type of blood sample (serum or plasma), type of infection (primary or secondary), dengue serotypes, and severity of disease (DF, DHF, DSS, or SD). Disagreements between the two reviewers were documented and resolved either through discussion with a third reviewer (B Khalid) or by contacting the authors for clarification. Data in the form of bar graphs or scatter plots (not in numeral values) were extracted by measuring the length of bars or by coordinating points using ImageJ software (National Institutes of Health, USA). Approximate values were then calculated.

### Data analysis and risk of bias (quality) assessment

Meta-analysis of mean differences was carried out using the OpenMeta[Analyst] software (Brown School of Public Health, Providence, RI, USA) and Comprehensive Meta-Analysis (CMA) V3.3.070 software (Biostat, Inc., Frederick, MD, USA), and the Meta-Essentials program (Erasmus Research Institute of Management, Netherlands). The I-squared index was used to assess heterogeneity between studies. Random effect and fixed effect models were used to calculate the mean effect size of studies with significant heterogeneity (*I*^2^ > 75%) and without significant heterogeneity (*I*^2^ < 75%), respectively. For studies that presented heterogeneity, subgroup analyses and one study omitting analysis were conducted to identify the source of heterogeneity. Publication bias analyses were carried out by visually observing for an asymmetrical funnel plot, as well as via Egger’s analysis, Begg’s analysis, Rosenthal’s fail-safe N, Orwin’s fail-safe N, and the trim and fill method (refer to Sheet 3 of  Supplemental Information 2). General agreement of results among these methods (at least four out of the six methods showed the presence of publication bias) was interpreted as sufficient evidence of publication bias (Ferguson, 2007).

Meta-analyses were carried out to combine biomarker levels of DF and SDI separately from all the studies. Combined mean levels of biomarkers during DF and SDI were compared. A *p*-value of <0.05 was interpreted as significantly different. To prevent the factor of days since fever onset from affecting the level of biomarkers, studies without stated days since onset of fever were then excluded when calculating the mean difference of biomarkers between DF and SDI. Only data that included days since onset of fever were used. If the number of days or intervals since fever onset matched, data for DF were compared with data for SDI from the same study, to calculate the mean difference. Data with the same number of days since fever onset were not combined if they came from different studies, as different studies may use different expressions (such as days of illness, days of fever, days of defervescence) and define the timing of measurement differently. Meta-analyses were carried out to combine all the mean differences from the selected studies of interest that reported the same number of days since fever onset. Subgroup analyses of mean differences in biomarker levels between DF and SDI patients according to WHO’s 1997 and 2009 classifications were conducted, as well as analyses separated by serum or plasma samples. Biomarker levels were compared among healthy control, DF, and SDI patients to determine if biomarker levels correlate with severity of dengue infection. Mean difference results from individual studies were compared with combined results to ascertain whether or not the results agree with one another.

Finally, subgroup analyses of mean biomarker levels were conducted separately for: (1) healthy control plasma and serum samples; (2) primary and secondary infections; and (3) infections caused by different dengue serotypes. For all subgroup analyses, a *p*-value of <0.05 was interpreted as significantly different.

### Definition and outcomes

Serum or plasma biomarkers were measured using flow cytometry or enzyme linked immunosorbent assay (ELISA) with an enzyme coupled antibody or fluorescent labeled antibody. DF, DHF, DSS, and SD were defined according to WHO’s classifications from 1997 and 2009. For this study, DHF, DSS, and SD are collectively known as SDI. Biomarkers refer to all chemokines, cytokines, and other proteins (Table 1). In contrast, severity markers refer to biomarkers that show a significant difference between DF and SDI and correlate with severity. Correlation with severity was defined as when biomarker levels increased or decreased in relation to severity (either in the order of SDI > DF > control, or SDI < DF < control). Timing of peak difference between DF and SDI was expressed in relation to days since onset of fever. Some studies used days of defervescence. As such, days of defervescence was converted into days since onset of fever, with the assumption that defervescence occurs on day 5 of the illness (Bachal et al., 2015). The outcome was defined as the effect size of difference in mean biomarker levels between DF and SDI patients. Raw mean difference was calculated according to the method described in a previous study (Borenstein et al., 2009).

## Results

### Results of literature search

**Figure 1:**
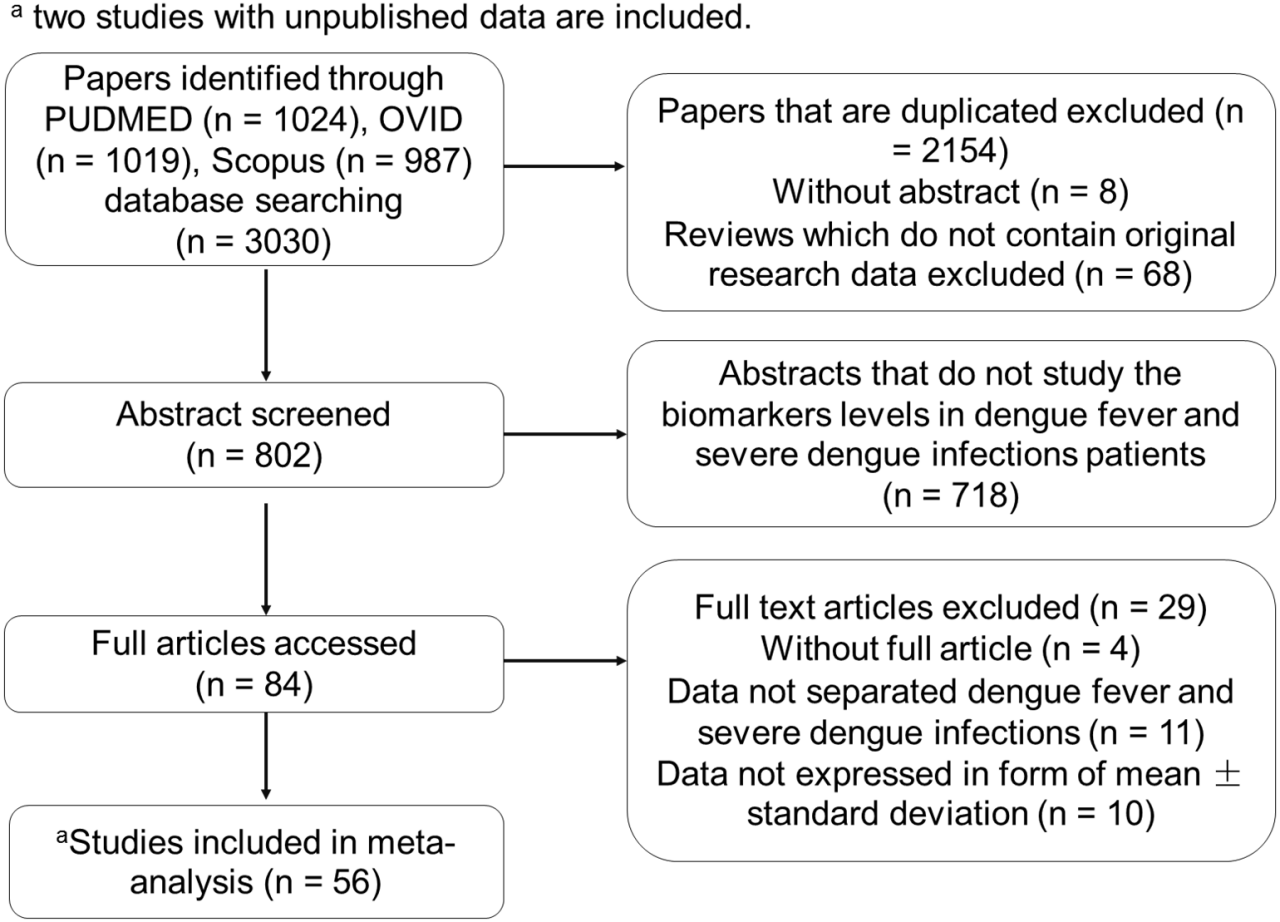
Flow chart of the study’s selection process.

The study’s selection process is depicted in Fig. 1. Fifty-six studies were included in this meta-analysis. The authors of forty-nine studies were contacted for unpublished data, and two sets of authors, Eppy et al. (2016) and De la Cruz Hernández et al. (2016), responded to provide their unpublished data. These two studies were also included. Detailed results of the literature search are provided in Sheet 11 ( Supplemental Information 2) for the benefit of future reviewers.

Table 2 presents the sample size, days of illness, study period, population, age, WHO classification method, and cytokines studies. All the selected studies were either cross-sectional or cohort studies.

**Table 2:** Characteristics of studies included in the meta-analysis.

No.	Study	Study population	Study duration	Time of experiment (days of fever)	WHO classification (year)	Sample size	Age of patients (years)
1	Kurane et al. (1991)	Thailand	1987–1988	1–20	1980	42	4–14
2	Yadav et al. (1991)	Malaysia	1989–1990	NA	1980	24	3–49
3	Kurane et al. (1993)	Thailand	NA	1–20	1986	112	5–14
4	Agarwal et al. (1999)	India	1996	1–18	NA	46	0.7–55
5	Green et al. (1999a)	Thailand	1994–1998	2–6	1986	37	NA
6	Green et al. (1999b)	Thailand	1994	1–60	1986	38	NA
7	Kittigul et al. (2000)	Thailand	1997	2–6	1997	22	<15
8	Braga et al. (2001)	Brazil	1997	1–5	PAHO 1994	30	NA
9	Murgue, Cassar & Deparis (2001)	French Polynesia	1996–1997	1–10	1997	230	0.1–15
10	Mustafa et al. (2001)	India	1996	1–18	1993	71	NA
11	Libraty et al. (2002)	Thailand	1994–1997, 1999–2000	1–6	NA	54	0.5–14
12	Koraka et al. (2004)	Indonesia	1995–1996	1–13	1997	58	0.6–14
13	Perez et al. (2004)	Cuba	1997	4–5	1997	26	16–59
14	Chen et al. (2005)	Taiwan	2002–2003	2–7	NA	99	20–81
15	Azeredo et al. (2006)	Brazil	2001–2003	1–10	2002	50	15–73
16	Butthep et al. (2006)	Thailand	NA	2–7	NA	86	4–16
17	Chen et al. (2006)	Taiwan	2002	1–18	1997	71	All ages
18	Khongphatthanayothin et al. (2006)	Thailand	NA	2.5–6.5	1997	60	NA
19	Lee et al. (2006)	Vietnam	NA	NA	NA	44	NA
20	Srikiatkhachorn et al. (2006)	Thailand	1994–2001	2–6	1993	99	NA
21	Chen et al. (2007)	Taiwan	2002–2003	2–7	NA	250	20–78
22	Wang et al. (2007)	Taiwan	2002–2003	NA	NA	69	15–80
23	Dejnirattisai et al. (2008)	Thailand	NA	2–11	2002	98	NA
24	Restrepo et al. (2008)	Colombia	2000–2002	1–5	PAHO 1995	34	NA
25	Valero et al. (2008)	Venezuela	NA	5–9	NA	32	1–52
26	Houghton-Trivino et al. (2009)	Colombia	2005–2006	2–7	2005	38	2–6
27	Levy et al. (2010)	Venezuela	2005–2006	NA	NA	70	3–53
28	Priyadarshini et al. (2010)	India	2005	2–15	1999	221	1–64
29	Ubol et al. (2010)	Thailand	NA	3–40	NA	30	NA
30	Voraphani et al. (2010)	Thailand	2005–2006	1–5	NA	23	<12
31	Furuta et al. (2012)	Vietnam	2002–2005	NA	1997	121	0.5–15
32	Kumar et al. (2012)	Singapore	2005–2006	2–7	1997	62	NA
33	De-Oliveira-Pinto et al. (2012)	Brazil	2007–2008	2–9	NA	43	21–63
34	Guerrero et al. (2013)	Colombia	NA	3–6	2009	89	NA
35	Jain et al. (2013)	India	NA	NA	2009	221	All
36	Malavige et al. (2013)	Sri Lanka	2011	4–5, 10–14	2011	259	NA
37	Arias et al. (2014)	Venezuela	NA	NA	2009	30	1–39
38	Limonta et al. (2014)	Brazil	2010	1–11	2009	63	NA
39	Misra, Kalita & Singh (2014)	India	2010	NA	NA	20	25–60
40	Soundravally et al. (2014)	India	NA	NA	1997	81	24–52
41	Vivanco-Cid et al. (2014)	Mexico	2010–2012	1–10	1997	152	5–74
42	Conroy et al. (2015)	Colombia	NA	1–7	1997	111	>5
43	Chunhakan et al. (2015)	Thailand	NA	3–7	NA	55	NA
44	Ferreira et al. (2015)	Brazil	2008	2–16	1997	52	0.2–14
45	Michels et al. (2015)	Indonesia	2011–2012	NA	2009	91	NA
46	Pandey et al. (2015)	India	2010–2012	2–5	2009	71	All ages
47	Patra et al. (2015)	India	2013–2014	NA	2011	40	NA
48	De la Cruz Hernández et al. (2016)^a^	Mexico	NA	0–5	1997	171	0–25
49	Eppy et al. (2016)^a^	Indonesia	2014–2015	3–5	2011	44	≥14
50	Fernando et al. (2016)	Sri Lanka	2015	3–9	2009	55	NA
51	Kamaladasa et al. (2016)	Sri Lanka	NA	3–7.5	2011	36	NA
52	Oliveira et al. (2016)	Brazil	NA	1–5	1997	77	NA
53	Senaratne, Carr & Noordeen (2016)	Sri Lanka	2011–2012	3–5	2011	67	NA
54	Singla et al. (2016)	India	2011	1–10	2009	43	4–14
55	Thakur et al. (2016)	India	NA	1–9	NA	106	NA
56	Zhao et al. (2016)	China	2013	3–17	2009	61	18–61

**Notes.**

NA represents information or data not available.

a represents contain unpublished data.

### Potential severity markers

In this meta-analysis, 2021 DF and 1728 SDI cases were analyzed. Nevertheless, only 357 DF and 474 SDI cases reported the number of days since fever onset (refer to Sheet 9 of  Supplemental Information 2).

**Figure 2:**
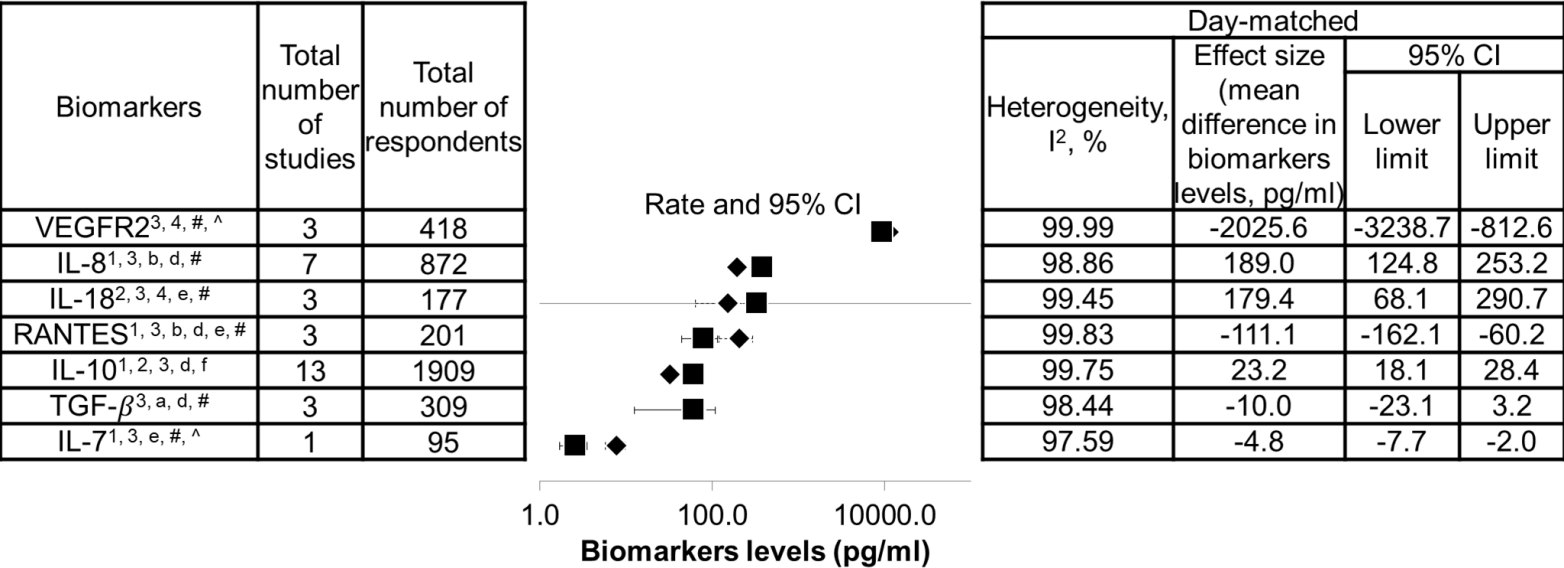
List of severity markers. Data are arranged in descending order of mean differences in biomarker levels. The positive criteria that qualified biomarkers as severity markers are represented by numbers (1–4), whereas negative criteria that disqualified biomarkers are represented by letter (a–f). Inadequate data are represented by symbols (*, #, ∧). Black diamond represents biomarkers levels in DF patients; black square represents biomarkers levels in SDI patients; ^1^ represents biomarkers that showed significant difference in levels between dengue fever (DF) and severe dengue infections (SDI) based on WHO 1997, WHO 2009, or both (*p* < 0.05); ^2^ represents biomarkers that showed significant difference in levels between DF and SDI in both serum and plasma samples; ^3^ represents biomarker levels that were correlated with severity; ^4^ represents an individual study that agreed with the combined results; ^a^ represents biomarkers that showed no significant difference in levels between DF and SDI, based on WHO 1997 and WHO 2009 (*p* > 0.05); ^b^ represents biomarkers that showed no significant difference between DF and SDI in either serum or plasma samples; ^c^ represents biomarker levels that did not correlate with severity; ^d^ represents an individual study that disagreed with the combined results; ^e^ represents the fact that no day-matched comparisons between DF and SDI were carried out; ^f^ represents the presence of publication bias, indicated by general agreement of results of funnel plot, Begg’s analysis or Egger’s analysis (*p* < 0.05), Rosenthal’s or Orwin’s fail safe *N* (*n* < 1000), trim and fill method (*p* < 0.05); ^#^ represents data not separated for dengue hemorrhagic fever and dengue shock syndrome, hence unable to perform subgroup analysis based on WHO 1997; ^*^ represents missing data for healthy control; unable to correlate biomarkers levels with severity; ^∧^ represents missing performance of one study omitting analysis and publication bias due to insufficient number of studies (total number of studies ≤ 2).

**Figure 3:**
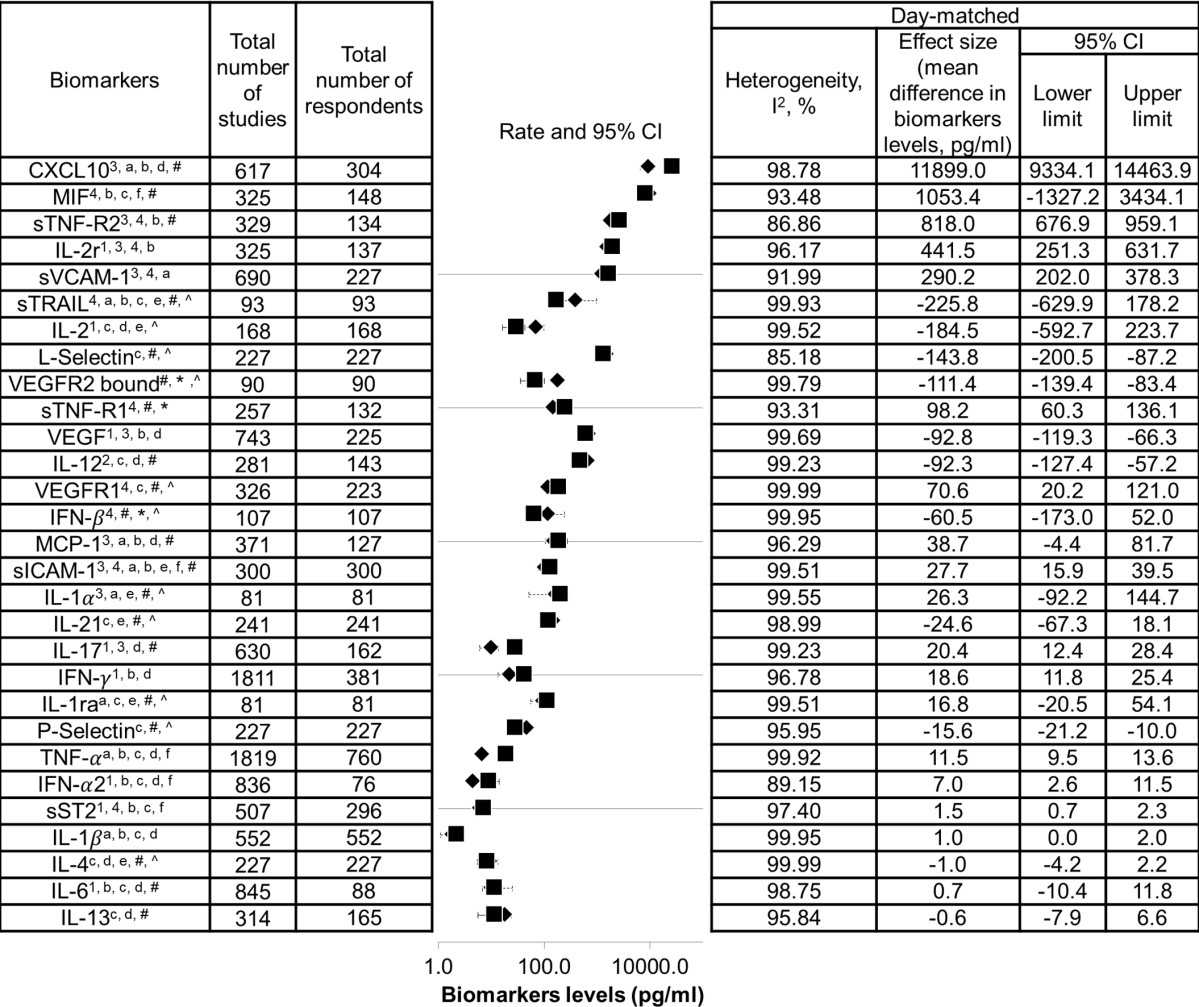
List of non-severity markers. Data are arranged in descending order of mean differences in biomarker levels. The positive criteria that qualified biomarkers as severity markers are represented by numbers (1–4), whereas negative criteria that disqualified biomarkers are represented by letter (a–f). Inadequate data are represented by symbols (*, #, ∧). Black diamond represents biomarkers levels in DF patients; black square represents biomarkers levels in SDI patients; ^1^ represents biomarkers that showed significant difference in levels between dengue fever (DF) and severe dengue infections (SDI) based on WHO 1997, WHO 2009, or both (*p* < 0.05); ^2^ represents biomarkers that showed significant difference in levels between DF and SDI in both serum and plasma samples; ^3^ represents biomarker levels that were correlated with severity; ^4^ represents an individual study that agreed with the combined results; ^a^ represents biomarkers that showed no significant difference in levels between DF and SDI, based on WHO 1997 and WHO 2009 (*p* > 0.05); ^b^ represents biomarkers that showed no significant difference between DF and SDI in either serum or plasma samples; ^c^ represents biomarker levels that did not correlate with severity; ^d^ represents an individual study that disagreed with the combined results; ^e^ represents the fact that no day-matched comparisons between DF and SDI were carried out; ^f^ represents the presence of publication bias, indicated by general agreement of results of funnel plot, Begg’s analysis or Egger’s analysis (*p* < 0.05), Rosenthal’s or Orwin’s fail safe *N* (*n* < 1000), trim and fill method (*p* < 0.05); ^#^ represents data not separated for dengue hemorrhagic fever and dengue shock syndrome, hence unable to perform subgroup analysis based on WHO 1997; ^*^ represents missing data for healthy control; unable to correlate biomarkers levels with severity; ^∧^ represents missing performance of one study omitting analysis and publication bias due to insufficient number of studies (total number of studies ≤ 2).

The list of biomarkers that exhibited significant differences between DF and SDI is shown in Fig. 2. Additionally, as these biomarkers were found to correlate with severity of dengue infection, they could also be potential severity markers. In contrast, a list of biomarkers that did not show significant difference between DF and SDI, did not correlate with severity, or both is presented in Fig. 3. These biomarkers will not be recommended as severity markers. Meta-analyses were not performed for some biomarkers (MIP-1 *β*, Eotaxin-1, CXCL9, CXCL11, IL-9, IL-18BP, IL-18FP, IL-33, E-selectin, MMP-2, and MMP-9) due to insufficient data.

Within the list of severity markers (Fig. 2), IL-8, IL-10, and IL-18 showed increased levels during SDI (95% CI, 18.1–253.2 pg/mL, 3–13 studies, *n* = 177–1,909, *I*^2^ = 98.86%–99.75%). Conversely, RANTES, IL-7, TGF-*β*, and VEGFR2 showed decreased levels during SDI (95% CI, –3238.7 to –3.2 pg/mL, 1–3 studies, n = 95–418, *I*^2^ = 97.59%–99.99%).As there was only one study that reported on IL-7, more studies are needed to further confirm its role as a potential severity marker. When subgroup analyses based on the 1997 and 2009 WHO classifications were conducted, it was found that most studies did not separate DHF and DSS cases. The mean difference between DHF or DSS and DF was therefore unable to be determined. For subgroup analyses of serum and plasma samples, the results of IL-10 and IL-18 showed significant differences between DF and SDI in both samples (refer to Sheet 2 of  Supplemental Information 2). Furthermore, when the results of individual studies were checked, results that disagreed with the combined results for RANTES, IL-8, IL-10, and TGF-*β* were discovered (see Fig. 2).

Sheet 3 of  Supplemental Information 2 shows detailed heterogeneity and publication bias analyses. High heterogeneity (*I*^2^ > 75%) was found in most meta-analyses performed in this study, except for biomarkers investigated by fewer studies. High heterogeneity (*I*^2^ > heterogeneity) was not reduced when one study omitting analyses and subgroup analyses according to WHO classification, as well as types of sample, host immune status, and serotypes, were conducted (see Sheet 3 of  Supplemental Information 2 ). Additionally,publication bias analyses indicated the presence of publication bias for IL-10 (see list of severity markers, Fig. 2), and TNF-*α*, MIF, IFN-*α*, and sICAM-1 (see list of non-severity markers, Fig. 3).

**Figure 4:**
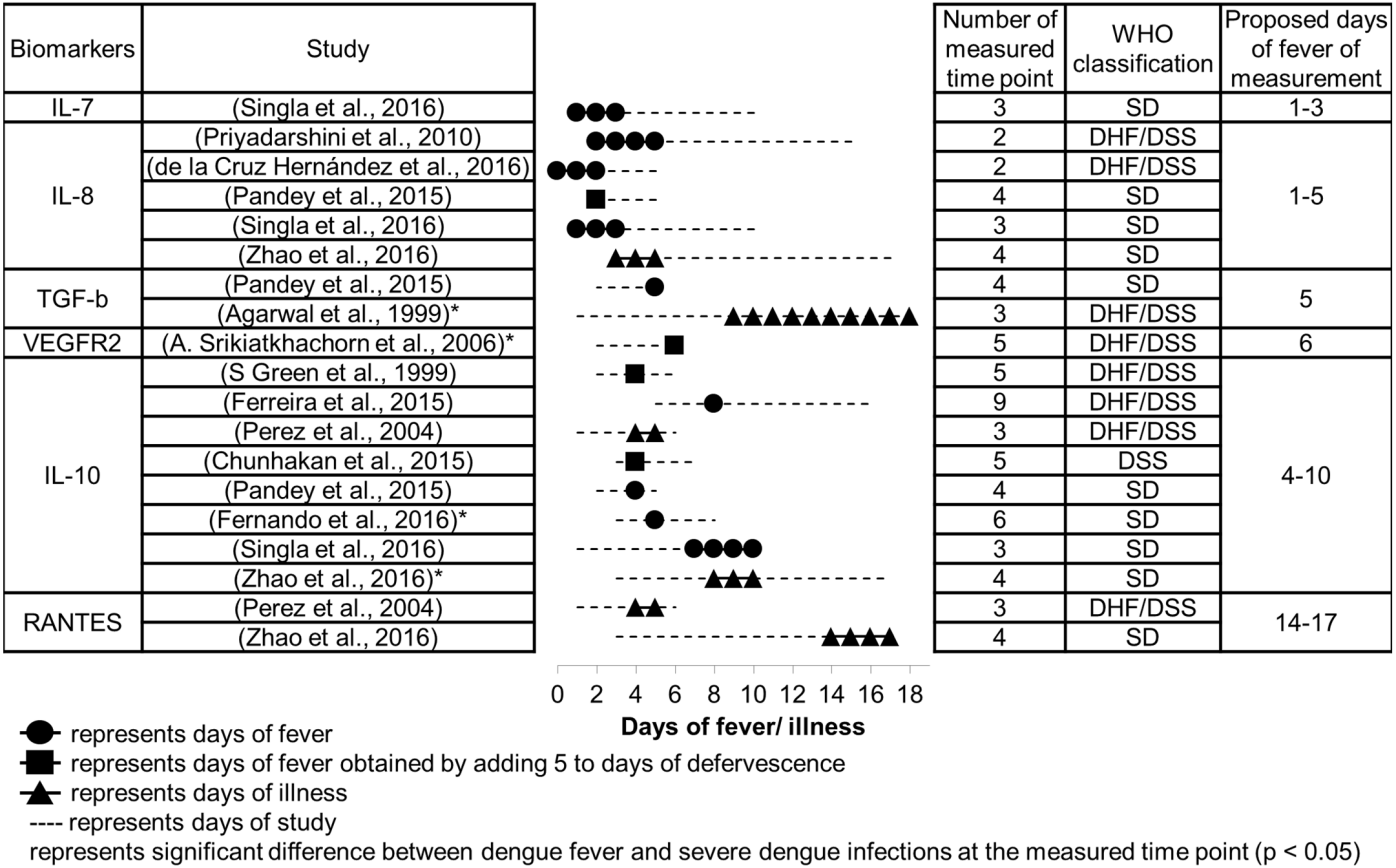
Days since fever onset at which the peak difference in biomarker levels between DF and SDI patients occurred.

### Suitable time to measure severity markers

The number of days since fever onset at which the difference in biomarker levels between DF and SDI patients were greatest are presented in Fig. 4. Proposed periods for fever measurement of these biomarkers are shown in the table. These proposed days for measurement are in accordance with several principles. First, priority was given to studies that used the 2009 WHO classification over those that used the 1997 WHO classification, because the 2009 WHO classification is better at representing severity (Horstick & Ranzinger, 2015). The manifestations of severe clinical symptoms were fewer in DHF, particularly DHF grade I and II as per the 1997 WHO classification, in comparison to symptoms manifested in SD, according to the 2009 WHO classification. Second, lower priority was given to studies with poor timing of sample collection (fewer measured time points and large intervals of measurements), as timing may obfuscate any difference in biomarker levels that may exist between different days after fever onset, as well as between patients with different severities. Finally, for some biomarkers where dispute in timing of peak response still existed among different studies after applying the above principles, ranges of days of measurement were combined to give a larger range.

Based on the meta-analysis, severity markers that are suitable to measure during the febrile phase (days 1–3) are IL-7 and IL-8 (Fig. 4). The biomarkers IL-8, IL-10, and TGF-*β* are suitable to measure during the critical phase (days 4–5), and VEGFR2 and IL-10 can be measured during the recovery phase (days 6–10). RANTES showed the greatest difference during a much later time than the recovery phase, days 14–17. No data were available concerning days of peak difference for IL-18.

### Subgroup analyses

Subgroup analyses were carried out to study associations between severity markers and the type of sample, host immune status, and dengue serotypes. The results of subgroup analyses of severity biomarkers showed that there are significant differences in IL-8 and VEGFR2 levels between serum and plasma samples (Table 3). IL-8 was significantly higher in serum samples, whereas VEGFR2 was significantly higher in plasma samples. For subgroup analyses of host immune status, IL-8 and IL-10 levels were significantly higher in secondary infections than in primary infections. Finally, subgroup analyses of dengue serotypes showed that there are overall significant differences in IL-8, IL-10, and TGF- *β* levels among different dengue serotypes (*p*-value of ANOVA <0.05), but no significant differences among individual dengue serotypes when paired comparisons were conducted (*p*-value of *t*-test >0.05; see Sheet 8 of  Supplemental Information 2 ). Moreover, subgroup analyses were unable to be performed for most severity markers due to an insufficient amount of studies and/or data. Thus, more research is needed to further confirm these findings.

**Table 3:** Results of subgroup analyses. Subgroup analyses of mean biomarker levels for: (1) healthy control of plasma and serum samples; (2) primary and secondary infections; and (3) infections caused by different dengue serotypes.

Biomarker	Type of sample (sera or plasma)			Host immune status (primary or secondary)			Dengue serotypes		
	Total number of studies	*n*	*p*-value	Total number of studies	*n*	*p*-value	Total number of studies	*n*	*p*-value
RANTES	2	19	0.065	2	106	0.248	NA	NA	NA
IL-7	1	NA	NA	1	75	0.596	1	8	0.175
IL-8	6	293	^*^Sera >plasma	3	427	^*^Secondary >primary	4	328	^*^0.000
IL-10	7	228	0.061	4	433	^*^Secondary >primary	3	161	^*^0.000
IL-18	2	32	0.143	NA	NA	NA	NA	NA	NA
TGF-*β*	1	NA	NA	1	211	0.400	1	69	^*^0.002
VEGFR2	3	64	^*^Plasma >sera	NA	NA	NA	NA	NA	NA

**Notes.**

NA, not available.

* Represents significant difference in biomarkers’ level in the subgroup analyses.

## Discussion

### Potential severity markers and when they should be measured

Meta-analysis results suggest that RANTES, IL-7, IL-8, IL-10, IL-18, TGF-*β*, and VEGFR2 could be potential severity markers for dengue infections (Fig. 2). Based on Sheet 10 ( Supplemental Information 2), when data were clustered according to different levels of severity (control, DF, and DHF/DSS for 1997 WHO classification; control, DF with/without warning signs, and SD for 2009 WHO classification), there was a significant difference in the level of IL-10 in relation to each level of severity. This suggests that IL-10 significantly correlates with severity. Meta-analyses of further separation between DHF and DSS cases, and between DF cases with and without warning signs were unable to be performed due to insufficient data.

**Figure 5:**
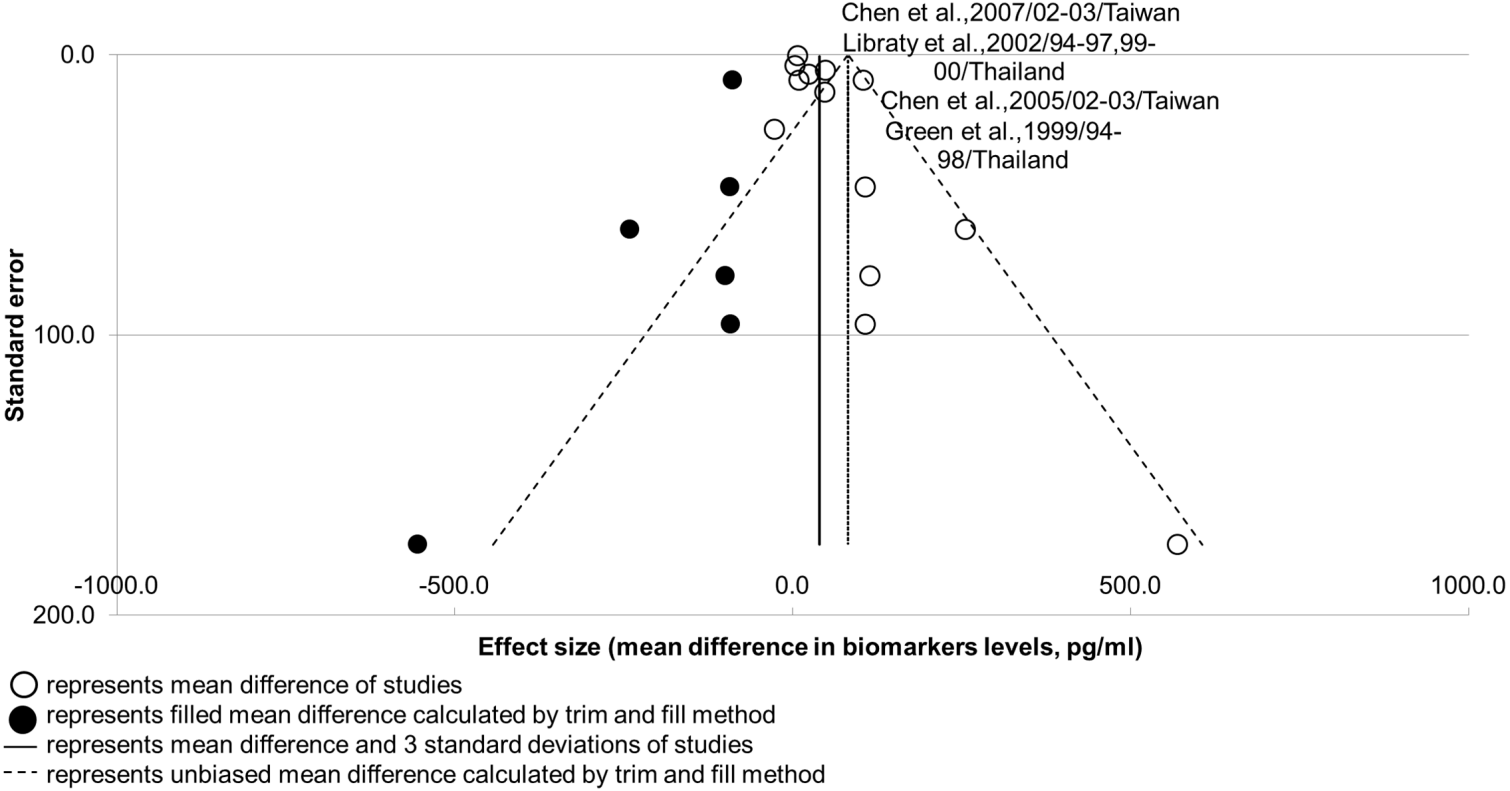
Funnel plot for potential biomarker IL-10. Shape of funnel plot showed obvious asymmetry. Unbiased mean difference calculated from trim and fill method was significantly different from the original estimate (*p*-value < 0.05). Two pair of studies presented with an overlap of five to seven co-authors, as well as study durations and locations.

Nevertheless, the results suggest that publication bias could be present in IL-10 studies (Fig. 2; see Sheet 3 of  Supplemental Information 2). The robustness of this analysis result was supported by a high number of studies (*n* > 10), but its power of analysis remains questionable with the presence of high heterogeneity (*I*^2^ > 75%; Field & Gillett, 2010; Rücker, Carpenter & Schwarzer, 2011). Publication bias could be present in IL-10 studies due to the unavoidable selection bias of patients from the same populations, evidenced by the overlap in co-authors, study duration, and locations in two pairs of studies. Publication bias would less likely to be caused by the time lag bias, as the low effect size of non-significant results did not correlate with the longer interval of publication (*r* < 0.50; see Sheet 3 of  Supplemental Information 2). A funnel plot for IL-10 is shown in Fig. 5.

Concerning course of infection, it was found that the majority of SDI (>70%) were recorded at day 3–6 of fever onset (Sheet 9 of  Supplemental Information 2). This resembles the critical phase (day 4–5 of fever onset) in the course of dengue infection, as defined by the Ministry of Health, Malaysia (Ministry of Health Malaysia & Academy of Medicine Malaysia, 2010). Severity markers that showed peak difference during or earlier than this phase—IL-7, IL-8, IL-10, TGF-*β*, and VEGFR2—could be potential early severity markers.

Nevertheless, some studies reported timing of measurement using different expressions, such as days of illness, days of fever, and days of defervescence, due to the different objectives of the studies (Fig. 4). Although days of defervescence were converted to days of fever in this meta-analysis based on the assumption that the first day of defervescence occurs on day 5 after fever onset (Bachal et al., 2015), the first day could in fact range from day 3 to day 7 (World Health Organization, 2009). Additionally, no response was received from authors concerning whether days of illness referred to days of fever or days of defervescence. Patients in those studies may also not have been aware of their days of fever or they may not have reported their experience of defervescence. This heterogeneity in methods, and failure to document the days of measurement or predict the timing of peak difference of severity biomarkers, would be prudent to overcome in future research before any conclusion can be made. It is also recommended that researchers should have a uniform definition of timing of measurement, perhaps days since fever onset. On clinical grounds, typical dengue infection has three phases: the febrile phase, the defeverscence phase, and the recovery phase. Definition of timing based on days since fever onset is linked with this typical course of dengue infection. In order to find biomarkers with higher levels to suggest prognostication, stricter criteria within this ideal condition are needed. Meanwhile, days of illness, which may or may not constitute complaint of fever by the patients, are strongly linked with atypical dengue presentation. This further obscures the targeted suitable timing for measuring severity biomarkers. Different methodologies could also be accepted, however, provided that information concerning days since fever onset is reported.

Results of the subgroup analyses of types of samples suggest that IL-8 could be more suitable to measure in serum samples, as it presents higher levels in such samples, whereas VEGFR2 could be more suitable to measure in plasma samples. Both these markers are also present with higher standard deviations, however, which causes them to be unstable as biomarkers in such samples. Researchers must therefore take careful consideration when choosing the type of sample to use. The cause for higher levels of VEGFR2 in plasma has yet to be explained, whereas it has been hypothesized that higher levels of IL-8 in sera are caused by activated platelets and leukocytes in the absence of clot factors in serum samples (Gruen et al., 2016). As such, levels of other severity biomarkers, such as IL-7, TGF-*β*, and RANTES, could also be higher in serum samples, as these biomarkers are released during platelet activation (De Azeredo, Monteiro & de Oliveira Pinto, 2015; Rathakrishnan et al., 2012; Wasilewska et al., 2005). In the current study, however, there were insufficient data to perform comparisons for these biomarkers.

### Immunopathogenesis of SDI

Levels of sICAM-1, sVCAM-1, and sST-2 did not show significant differences between DF and SDI patients (Fig. 3). These biomarkers are indicators of endothelial activation or damage (Butthep et al., 2006); therefore, the results suggest that endothelial damage or activation may be similar in both DF and SDI, and endothelial damage may not play a significant role in causing SDI. This provides evidence to strengthen the role of soluble vasoactive factors.

The results showed that IL-8, IL-18, and IL-10 levels increased during SDI, whereas RANTES, IL-7, VEGFR2, and TGF-*β* levels decreased (Fig. 2). Previous studies have disclosed that dengue virus-induced endothelial cells release more IL-8, but less soluble VEGFR2 (Srikiatkhachorn et al., 2006; Srikiatkhachorn & Green, 2010). IL-8 acts synergistically with TNF- *α* to disrupt tight junctions among endothelial cells, increasing endothelial permeability (Bozza et al., 2008; Kelley, Kaufusi & Nerurkar, 2012). Conversely, a decrease in soluble VEGFR2 in the blood increases the expression of certain receptors on the endothelial surface and their binding with VEGF. VEGF-VEGFR2 signaling affects adherens junctions and tight junctions, further increasing endothelial permeability (Steinberg, Goldenberg & Lee, 2012; Van de Weg et al., 2014).

**Figure 6:**
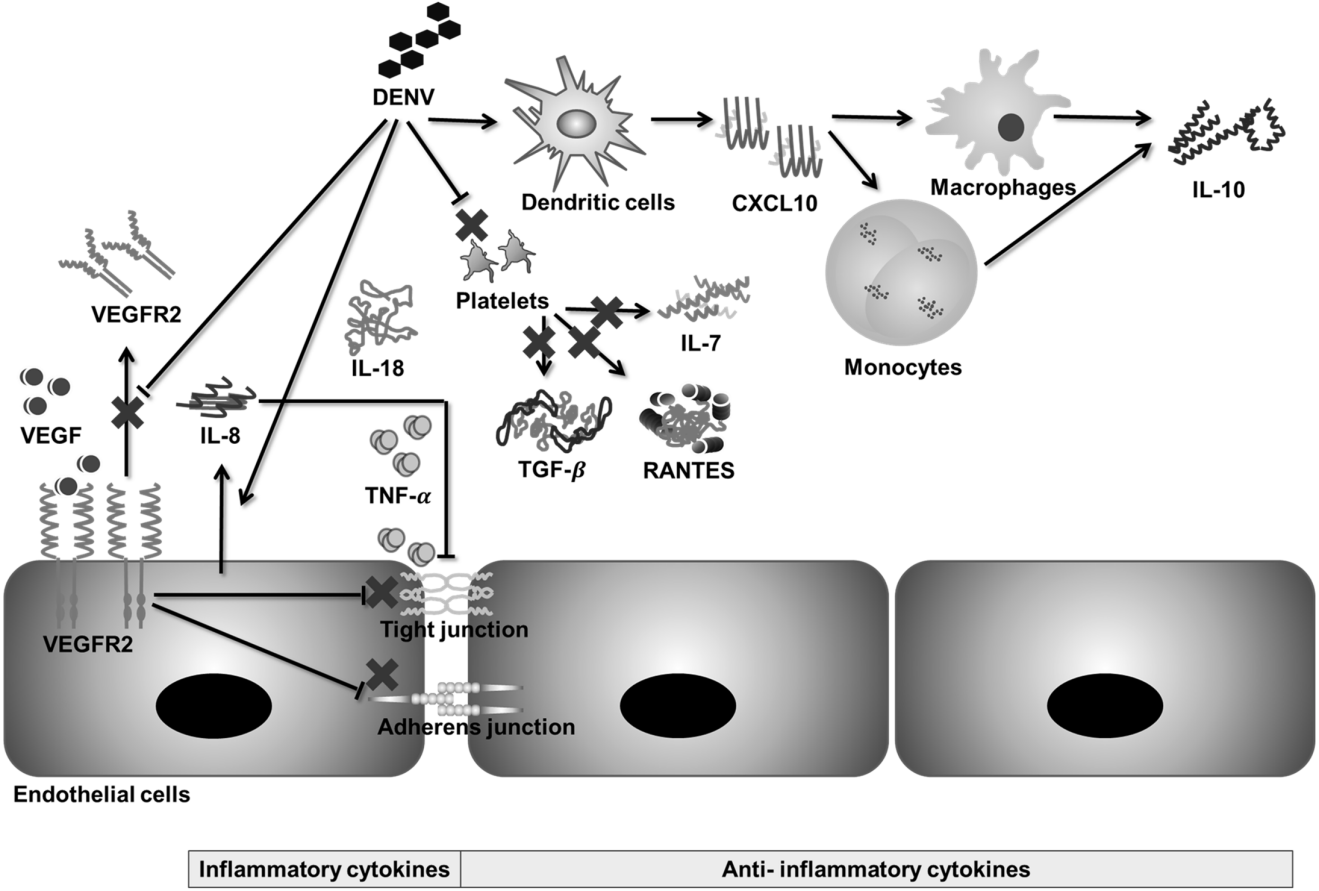
Hypothetical pathway for immunopathogenesis of dengue infection. During severe dengue infection (SDI), levels of soluble VEGFR2, TGF-*β*, IL-7, and RANTES are suppressed, whereas levels of IL-8, IL-18, and IL-10 increase significantly. VEGF-VEGFR2 signaling, IL-8, and TNF-*α* increase endothelial permeability by disrupting tight junctions and adherens junctions between endothelial cells. Changes in levels of IL-18, IL-7, TGF-*β*, RANTES, and IL-10 are related to prolonged inflammation during SDI.

RANTES, IL-7, and TGF-*β* are released during platelet activation (De Azeredo, Monteiro & de Oliveira Pinto, 2015; Rathakrishnan et al., 2012; Wasilewska et al., 2005); therefore, a decrease in these biomarkers during SDI could be due to thrombocytopenia, as these biomarkers function to suppress inflammation and viral clearance (Azeredo et al., 2006). Furthermore, a decrease in these biomarkers causes prolonged inflammation and increased permeability (Rathakrishnan et al., 2012; Singla et al., 2016). In contrast, IL-10 has been found to positively correlate with platelet decay during dengue infection (Perez et al., 2004). The main sources of IL-10 release are virus immune complex infected macrophages, memory cells, and infected monocytes (Perez et al., 2004), which may explain the observation that IL-10 does not decrease with thrombocytopenia. Moreover, administration of IL-10 does not cause adverse effects in the human body (Green et al., 1999), which suggests that IL-10 may act as an indicator that reflects severity rather than being a cause of severity. Moreover, a pro-inflammatory cytokine, IL-18, has also been found to be associated with sepsis by increasing endothelial permeability (Azeredo et al., 2006). Based on the findings in this meta-analysis and previous literature, a hypothetical mechanism of immunopathogenesis is illustrated in Fig. 6.

There was no significant difference in levels of CXCL10 and TNF-*α* between DF and SDI, but the difference was significant between control and DF (Sheet 10 of Supplemental Information 2). This result suggests that although CXCL10 and TNF-*α* may not be suitable severity biomarkers, they are involved in the immunopathogenesis of dengue infection in an antibody-independent manner (Sheet 7 of Supplemental Information 2).

Moreover, levels of IL-8 and IL-10 are significantly higher in secondary infections (Table 3), suggesting that these two cytokines play roles in the antibody-dependent enhancement mechanism. Nonetheless, there was insufficient data to evaluate the effect of previous exposure to dengue virus on levels of RANTES, IL-7, IL-18, TGF-*β*, and VEGFR2.

Based on the results of the subgroup analyses of dengue serotypes (Table 3), although there are overall significant variation among different dengue serotypes (*p*-value of ANOVA <0.05), no significant differences among individual dengue serotypes when paired comparisons were conducted (*p*-value of *t*-test >0.05; see Sheet 8 of  Supplemental Information 2). Therefore, this suggests that the severity biomarkers can be applied for dengue infections caused by different dengue serotypes, but requires careful consideration. In relation to non-severity biomarkers, it was found that dengue-3 significantly induced higher levels of IL-12 and sVCAM-1, whereas dengue-1 significantly induced lower levels of TNF-*α*, compared to other dengue serotypes. This indicates that different dengue serotypes could have different mechanisms of immunopathogenesis, which demands future research.

Throughout this study, it was found that biomarker levels vary significantly among different subgroups separated according to the WHO classification system, types of samples, host immune status, and dengue serotypes. Furthermore, heterogeneity was not reduced when these subgroup analyses were carried out. This strengthens the idea that the heterogeneity of biomarker levels was caused by multiple factors, rather than caused by any single factor, which explains the inconsistency of results in a previous review (Huy et al., 2013).

### Limitations

Although several severity markers were suggested in this meta-analysis study, readers are reminded that there are limitations to the data used. First, there are more biomarkers or MESH terms for biomarkers, that were unable to be covered here. Second, although we conducted subgroup analyses to analyze the effects of different factors on biomarker levels, the transient nature of the biomarkers can be affected by numerous other factors not covered in this meta-analysis, such as age, genetic composition, and clinical features of respondents, half-lives of cytokines, and fluid management of patients. This shows the need for future research that controls for these factors, in order to further confirm the findings.

Additionally, as this study defined severity markers as biomarkers that showed significant differences between DF and SDI over all days after fever onset, biomarkers that showed significant variation only on certain days of fever were neglected. Their applications as severity biomarkers are limited due to their transient nature. Because of this, their roles in the immunopathogenesis of dengue infection are beyond the scope of this study.

Finally, studies concerning the role of viremia as a marker for SDI are also beyond the scope of this meta-analysis. A study by Vaughn et al. (2000), which compared viremia using peak viremia titer, showed that increased dengue disease severity correlated with higher peak viremia titer and secondary infection patients possessed higher peak viremia titers. Nonetheless, the results displayed in Tables 2 and 3 of Sheet 10 (Supplemental Information 2) show that viremia was higher in DF in one study (Singla et al., 2016) and higher in SDI in other studies (Chen et al., 2005; Libraty et al., 2002). When primary and secondary infections patients were compared, viremia was found to be higher in primary infections in one study (Sung et al., 2016), and higher in secondary infections in another study (Singla et al., 2016). This suggests that although SDI and secondary infection patients have higher peak viremia titers, the rate of viremia clearance is also rapid (Tables 2 and 3 of Sheet 10 of Supplemental Information 2), which thereby causes the difference between DF and SDI to be smaller, or produces conflicting results. The results of this pilot study suggest that viremia is not suitable as a severity marker, therefore it is not discussed in this meta-analysis.

## Conclusions

Potential severity biomarkers—RANTES, IL-7, IL-8, IL10, IL-18, TGF-*β*, and VEGFR2—were presented in this study. Analysis of biomarker levels in relation to days since fever onset suggested that IL-7, IL-8, IL-10, TGF-*β*, and VEGFR2 are potential early biomarkers of SDI. Hence, this study provides possible severity markers to be used for reference in clinical practice, or in the development of immunomodulating drugs or inhibitors of severe inflammatory mediators.

##  Supplemental Information

10.7717/peerj.3589/supp-1 Supplemental Information 1 Prisma 2009 checklist

10.7717/peerj.3589/supp-2 Supplemental Information 2 Supporting information

10.7717/peerj.3589/supp-3 Supplemental Information 3 The rationale for conducting the meta-analysis
