# Supplementary material for: Meta-analysis of biomarkers for severe dengue infections
Source: PeerJ. 2017 Sep 15;5:e3589. doi: 10.7717/peerj.3589 (PMC5602679; doi:10.7717/peerj.3589)
Supplement: Supplemental Information 3 [file peerj-05-3589-s003.docx]

1. The rationale for conducting the meta-analysis.
2. The contribution that the meta-analysis makes to knowledge in light of previously published related reports, including other meta-analyses and systematic reviews.

Numerous studies (Kelley, Kaufusi, & Nerurkar, 2012; Reis et al., 2007; Seet et al.; Tay & Tan, 2006) and reviews (Chaturvedi et al. 2000; Huy et al. 2013; John, Lin, and Perng 2015; Page and Liles 2013; Yacoub et al. 2014) were conducted to propose potential severity biomarkers and timing of peak response. Nevertheless, one of the major hindrances of this effort is the inconsistency of results caused by heterogeneity among the studies. Level of biomarkers were found to be affected by factors such as timing of sample collection, processing of samples into plasma or serum, WHO classification method used to assign the disease’s severity, host’s immune status, and dengue serotypes (Srikiatkhachorn & Green, 2010). Therefore, this study aims to identify potential severity biomarkers and to study the factors causing inconsistency.

This study fills the gap in literature by performing meta-analysis on difference in levels of 53 biomarkers between healthy control, dengue fever and severe dengue infection in humans. Timing of sample collection in context of days of onset of fever was recorded with detailed subgroup analyses of (1) 1997 and 2009 WHO classifications, (2) plasma and serum samples, (3) patients’ antibody dependent enhancement status in context of dengue infection (primary and secondary infections) and (4) dengue serotypes were carried out. The outcome allows the identification of potential best severity biomarker and proposes the suitable timing to measure these severity markers.

Chaturvedi, U. C., R. Agarwal, E. A. Elbishbishi, and A. S. Mustafa. 2000. “Cytokine Cascade in Dengue Hemorrhagic Fever: Implications for Pathogenesis.” *FEMS immunology and medical microbiology* 28(3):183–88. Retrieved September 3, 2015 (http://www.ncbi.nlm.nih.gov/pubmed/10865168).

Huy, Nguyen Tien et al. 2013. “Factors Associated with Dengue Shock Syndrome: A Systematic Review and Meta-Analysis” edited by Scott B. Halstead. *PLoS Neglected Tropical Diseases* 7(9):e2412. Retrieved December 9, 2016 (http://www.ncbi.nlm.nih.gov/pubmed/24086778).

John, Daisy Vanitha, Yee-Shin Lin, and Guey Chuen Perng. 2015. “Biomarkers of Severe Dengue Disease – a Review.” *Journal of Biomedical Science* 22(1):83. Retrieved December 6, 2016 (http://www.ncbi.nlm.nih.gov/pubmed/26462910).

Kelley, JF, PH Kaufusi, and VR Nerurkar. 2012. “Dengue Hemorrhagic Fever-Associated Immunomediators Induced via Maturation of Dengue Virus Nonstructural 4B Protein in Monocytes Modulate Endothelial Cell Adhesion Molecules and Human Microvascular Endothelial Cells Permeability.” *virology* 422(2):326–37.

Page, Andrea V, and W. Conrad Liles. 2013. “Biomarkers of Endothelial Activation/dysfunction in Infectious Diseases.” *Virulence* 4(6):507–16. Retrieved September 7, 2015 (http://www.ncbi.nlm.nih.gov/pubmed/23669075).

Reis, Sônia Regina Nogueira Ignácio et al. 2007. “An in Vitro Model for Dengue Virus Infection That Exhibits Human Monocyte Infection, Multiple Cytokine Production and Dexamethasone Immunomodulation.” *Memórias do Instituto Oswaldo Cruz* 102(8):983–90. Retrieved October 2, 2015 (http://www.ncbi.nlm.nih.gov/pubmed/18209938).

Seet, Raymond C. S. et al. “Oxidative Damage in Dengue Fever.” *Free Radical Biology and Medicine* 47(4):375–80. Retrieved October 20, 2015 (http://www.ncbi.nlm.nih.gov/pubmed/19427377).

Srikiatkhachorn, Anon, and Sharone Green. 2010. “Markers of Dengue Disease Severity.” *Current topics in microbiology and immunology* 338:67–82.

Tay, Joc Cing, and Philip Tan. 2006. “Finding Intervention Points in the Pathogenesis of Dengue Viral Infection.” Pp. 5315–21 in *2006 International Conference of the IEEE Engineering in Medicine and Biology Society*, vol. 1. IEEE. Retrieved October 9, 2015 (http://www.ncbi.nlm.nih.gov/pubmed/17945891).

Yacoub, Sophie et al. 2014. “Predicting Outcome from Dengue.” *BMC Medicine* 12(1):147. Retrieved December 9, 2016 (http://bmcmedicine.biomedcentral.com/articles/10.1186/s12916-014-0147-9).
